# Alexithymia and anhedonia in early Richardson's syndrome and progressive supranuclear palsy with predominant parkinsonism

**DOI:** 10.1002/brb3.1448

**Published:** 2019-11-19

**Authors:** Francesca Assogna, Clelia Pellicano, Luca Cravello, Cinzia Savini, Lucia Macchiusi, Mariangela Pierantozzi, Alessandro Stefani, Bruno Mercuri, Carlo Caltagirone, Francesco E. Pontieri, Gianfranco Spalletta

**Affiliations:** ^1^ Fondazione Santa Lucia IRCCS Roma Italy; ^2^ UOC Neurologia Azienda Ospedaliera “Belcolle” Viterbo Italy; ^3^ Centro Alzheimer ASST Rhodense Rho Italy; ^4^ Dipartimento di Medicina dei Sistemi Università “Tor Vergata” Roma Italy; ^5^ UOC Neurologia Azienda Ospedaliera “San Giovanni Addolorata” Roma Italy; ^6^ Dipartimento di Neuroscienze Salute Mentale e Organi di Senso “Sapienza” Università di Roma Roma Italy

**Keywords:** alexithymia, anhedonia, nonmotor symptoms, Parkinson's disease, progressive supranuclear palsy

## Abstract

**Introduction:**

Phenotypic variants of progressive supranuclear palsy (PSP) are all characterized by the combination of motor symptoms of parkinsonism with a number of neuropsychiatric and cognitive disorders. Despite the strong effort in characterizing these features in PSP, alexithymia and anhedonia have not been investigated at present. Here, we aimed at investigating the qualitative and quantitative differences of alexithymia and anhedonia in the two more frequent variants of PSP, Richardson's syndrome (PSP‐RS) and PSP with predominant parkinsonism (PSP‐P) compared to Parkinson's disease (PD) patients recruited within 24 months after the onset of motor symptoms.

**Methods:**

One hundred fifty‐five PD, 11 PSP‐P, and 14 PSP‐RS patients underwent clinical, neuropsychiatric, and neuropsychological evaluations. Alexithymia was assessed using the Toronto Alexithymia Scale‐20 item (TAS‐20), and hedonic tone was measured by the Snaith–Hamilton Pleasure Scale (SHAPS).

**Results:**

In PSP‐P and PSP‐RS patients, the frequency of alexithymia diagnosis was higher than in PD. On the TAS‐20 scores, PSP‐RS performed worse in the total score and in F2 sub‐scale when compared to PD. Among patients with diagnosis of depression, PSP‐RS showed higher scores in TAS‐20 total and TAS‐20 F2 than PD. No significant differences in TAS‐20 scores were found in nondepressed patients. Finally, we did not find significant differences among PD, PSP‐P, and PSP‐RS groups in the SHAPS scores.

**Conclusions:**

Alexithymia is identifiable very early in PSP‐P and PSP‐RS patients. Alexithymic symptoms differentiate PSP‐RS group from PD group but not between the two subtypes of PSP. Further, alexithymia in PSP seems to be predicted by the presence of depression. Altered emotional capability could be related to specific neurophysiological dysfunction occurring precociously in PSP; therefore, its identification could orient the diagnosis toward PSP cases.

## Graphical abstract

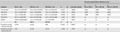

## 1 INTRODUCTION

Progressive supranuclear palsy (PSP) is the most common degenerative atypical parkinsonism (Litvan et al., 1996). The clinical spectrum of PSP is rather heterogeneous, with several variants being identified by the recent diagnostic criteria of the Movement Disorder Society (Hoglinger et al., 2017). Phenotypic variants of PSP are all characterized by the combination of motor symptoms of parkinsonism with a number of neuropsychiatric and cognitive disorders and other symptoms (pain, gastrointestinal impairment, fatigue, and sleep disorders). Richardson's syndrome (PSP‐RS) and PSP with predominant parkinsonism (PSP‐P) are the most frequent phenotypes (Hoglinger et al., 2017) of PSP. Motor and nonmotor symptoms of PSP produce significant disability and have severe negative impact on health‐related quality of life of patients and caregivers (Colosimo et al., 2010).

Despite the strong effort in characterizing the neuropsychiatric and neuropsychological features of the different variants of PSP, alexithymia and anhedonia have not been investigated at present. Alexithymia refers to the inability of recognizing and verbalizing emotions and distinguishing emotional states from bodily sensations and emotional arousal, whereas anhedonia is defined as the lowered ability to experience physical or social pleasure. Emotional processing and hedonic state modulate behavior by regulating mechanisms of motivation, promoting transgenerational relationships, and increasing chances to adapt to communities. Alexithymia and anhedonia have been reported frequently in patients suffering from Parkinson's disease (PD); however, it is still not clear whether they are a secondary phenomenon linked to depression and apathy severity (Assogna, Cravello, Caltagirone, & Spalletta, 2011; Assogna et al., 2016) or a disease primary characteristic (Assogna et al., 2011, 2016; Spalletta et al., 2013) linked to frontal lobe dysregulation.

Although frontal–subcortical dysfunction plays a relevant role in PSP neuropathology (Brenneis et al., 2004), the lack of reports on the frequency and severity of alexithymia and anhedonia in PSP patients prevents comparisons between PSP variants and among these variants and PD.

On the bases of this background, the aim of the study was to analyze qualitative and quantitative differences of alexithymia and anhedonia in PSP‐RS and PSP‐P compared to PD patients, early in the disease course.

## 2 METHODS

Twenty‐five PSP patients and 155 PD patients, aged 40–80 years, were consecutively enrolled at our Outpatient Services for Movement Disorders. In all these subjects, onset of motor symptoms dated <24 months at enrollment. Clinical diagnosis of PD or PSP was posed prospectively, after at least 3 years of follow‐up, according to the criteria by Gelb, Oliver, and Gilman (1999) and Hoglinger et al. (2017), respectively. In our sample, 11 cases of PSP‐P and 14 cases of PSP‐RS were identified (Hoglinger et al., 2017; Williams & Lees, 2009), as previously published by our group (Pellicano et al., 2017).

Exclusion criteria were comorbidity with major, not stabilized, medical illnesses; known or suspected history of alcoholism, drug dependence or abuse; other neurological disorders; head trauma with loss of consciousness; psychiatric disorders (apart from mood and anxiety disorders) diagnosed according to the Diagnostic and Statistical Manual of Mental Disorders, 4th Edition—Text Revision; brain tumors; and significant cerebrovascular pathology identified by CT or MRI scan. The amount of dopamine replacement therapy was calculated and expressed as daily levodopa equivalents, as previously published (Pellicano et al., 2017). Our experimental protocol was approved by the Ethical Committee of the Fondazione Santa Lucia, IRCCS, and each subject signed the informed consent before enrollment.

Within 2 weeks from enrollment, each participant underwent a complete clinical, neuropsychiatric, and neuropsychological evaluation. In particular, all subjects were submitted to a Structured Clinical Interview‐Patient Edition for identification of major depressive disorder and minor depressive disorder, according to the DSM‐IV‐TR criteria. A senior psychiatrist (GS) did this diagnostic assessment. Moreover, severity of depressive symptoms was investigated by the Beck Depression Inventory (total, psychic, and somatic scores). Detailed description of the evaluations has been previously reported by Pellicano and collaborators (Pellicano et al., 2017). To the aim of the present research, we report herein findings of an additional neuropsychiatric evaluation investigating alexithymia and anhedonia.

Alexithymia was assessed by the 20‐item Toronto Alexithymia Scale (TAS‐20; Bagby, Parker, & Taylor, 1994; Bagby, Taylor, Parker, & Loiselle, 1990). The TAS‐20 is a self‐report instrument with good internal consistency and reliability as well as construct and criterion validity for the measurement of alexithymia features. It comprises three subscales assessing different facets of alexithymia: F1, difficulty in identifying feelings; F2, difficulty in describing feelings; and F3, an externally oriented analytic mode of thinking. To evaluate the prevalence of alexithymia, patients with a TAS‐20 score > 60 were considered as alexithymic, those scoring 52–60 as borderline, and those scoring < 52 as non alexithymic.

Hedonic tone was investigated by the Snaith–Hamilton Pleasure Scale (SHAPS; Snaith et al., 1995), a self‐rated instrument consisting of 14 items that cover domains of social interaction, food and drink, sensory experiences, achievements, and pastimes. Subjects were requested to agree or disagree with a statement for each item on a liking scale (definitely agree, agree, disagree, and definitely disagree). The four available answers were divided into dichotomous categories (agree = 0; disagree = 1). Score ranged, therefore, from 0 to 14, with a cut‐off at 2 as the best discrimination between “normal” (score ≤ 2, categorized as hedonic) and “abnormal” (score > 2, categorized as anhedonic) level of hedonic tone.

Assessments were performed by three trained neuropsychologists (FA, CS, LM). Acceptable inter‐rater reliability was defined as *k* > 0.80.

Differences in demographic, clinical, neuropsychiatric, and neuropsychological features among groups were assessed by the chi‐square test for categorical variables and by a series of Kruskal–Wallis *H* tests for continuous variables followed by Mann–Whitney *U* test post hoc comparisons when appropriate. After Bonferroni's correction for multiple comparisons (*n* = 4; i.e., TAS‐20 F1, TAS‐20 F2, TAS‐20 F3, SHAPS), the level of statistical significance was defined as *p* < .05/4 = *p* < .0125.

Several lines of evidence suggest that depression and alexithymia are closely interrelated (Parker, Bagby, & Taylor, 1991). In order to take into account this relationship, in an ancillary analysis we split our sample by presence/absence of depression diagnosis, and we analyzed differences in TAS‐20 scores in patients with or without depression by a series of Kruskal–Wallis H tests followed by Mann–Whitney U test post hoc comparisons when appropriate. Given the exploratory nature of these last analyses, correction for multiple comparisons was not applied and the level of statistical significance was accepted as *p* < .05.

## 3 RESULTS

Two PSP‐RS patients were excluded from the original cohort because they did not complete assessment. Consequently, our cohort was composed by 155 PD, 11 PSP‐P, and 12 PSP‐RS subjects.

As to the severity of parkinsonian symptoms, PSP‐RS patients had significantly higher UPDRS‐III score as compared to PD patients (Table 1).

**Table 1:** Demographic and clinical characteristics of patients with PD, PSP‐P, and PSP‐RS

Characteristics	PD (*n* = 155)	PSP‐P (*n* = 11)	PSP‐RS (*n* = 14)	*H*	*df*	*p *(Kruskal–Wallis)	Post hoc analysis (Mann–Whitney *U* test)		
							*p* PD vs. PSP‐P	*p* PD vs. PSP‐RS	*p* PSP‐P vs. PSP‐RS
Age (years)	67.2 ± 7.47 (91.197)	65.4 ± 6.6 (69.273)	68.9 ± 7.5 (99.464)	2.268	2	.3218	na	na	na
Disease duration (months)	15.3 ± 8.4 (88.971)	15.3 ± 9.4 (89.682)	18.75 ± 5.2 (108.071)	1.728	2	.4214	na	na	na
Education (years)	10.5 ± 4.5 (90.926)	11.9 ± 5.2 (107.136)	8.8 ± 3.9 (72.714)	2.763	2	.2512	na	na	na
UPDRS‐III score	15.9 ± 9.3 (82.224)	21.2 ± 11.3 (107.944)	25.0 ± 6.5 (134)	10.184	2	.006*	.127	.004	.229
BDI total score	8.4 ± 6.4 (86.210)	10.5 ± 5.2 (114.045)	13.6 ± 9 (119.500)	7.634	2	.022*	.08	.02	.584
BDI psychic score	4.9 ± 4.5 (85.723)	6.7 ± 3.6 (116.409)	8.4 ± 5.7 (123.036)	9.481	2	.009*	.054	.011	.529
BDI somatic score	3.4 ± 2.6 (88.010)	3.8 ± 2.1 (100.182)	5.1 ± 4 (110.464)	2.789	2	.248	na	na	na
	**PD** **(*n* = 155)**	**PSP‐P** **(*n* = 11)**	**PSP‐RS** **(*n* = 14)**	***χ*^2^**	***df***	***p***			
Sex (male, %)	84 (54.2%)	7 (63%)	6 (42.8%)	1.117	2	.573	na	na	na
Apathy (Yes) (% Yes)	4 (2.6%)	3 (27.3%)	3 (21.4%)	19.225	2	<.0001*	na	na	na
Depression (Yes) (% Yes)	51 (32.9%)	7 (63.6%)	9 (64.3%)	8.91	2	.012*	na	na	na

Note

Data represent mean ± *SD* (Mean Rank).

Abbreviations: na, Not applicable; PD, Parkinson's disease; PSP‐P, progressive supranuclear palsy with predominant parkinsonism; PSP‐RS, progressive supranuclear palsy–Richardson's syndrome; UPDRS‐III, Unified Parkinson's Disease Rating Scale‐Part III.

* Significant at *p* < .05.

The three groups differed significantly in the TAS‐20 score. In particular, PSP‐RS patients scored worse than PD ones at the TAS‐20 total and F2 scores (Table 2). There were no significant differences of the SHAPS score among PD, PSP‐P, and PSP‐RS (Table 2). Finally, diagnosis of alexithymia but non anhedonia was more frequent in either PSP subtype with respect to PD (Table 2).

**Table 2:** Alexithymia and anhedonia in patients with PD, PSP‐P, and PSP‐RS

Variables	PD (*n* = 155)	PSP‐P (*n* = 11)	PSP‐RS (*n* = 12)	*H*	*df*	*p* (Kruskal–Wallis)	Post hoc analysis (Mann–Whitney *U* test)		
							*p* PD vs. PSP‐P	*p* PD vs. PSP‐RS	*p* PSP‐P vs. PSP‐RS
TAS‐20 total	46.2 ± 11.7 (84.074)	55.2 ± 18.1 (115.091)	59.2 ± 11 (136.125)	14.257	2	.0008*	.0662	.0005	.9264
TAS‐20 F1	12.2 ± 5.8 (85.787)	16.4 ± 7 (120.500)	17.6 ± 11.1 (109.042)	6.512	2	.0385	.0287	.1394	.8055
TAS‐20 F2	13.4 ± 5.9 (85.239)	16.4 ± 7.6 (108.636)	18.7 ± 7.1 (127.000)	8.933	2	.0115*	.1380	.0074	.2679
TAS‐20 F3	20.5 ± 5.6 (87.587)	22.4 ± 9.5 (90.318)	22.9 ± 7.3 (113.458)	2.811	2	.2453	na	na	na
SHAPS	0.439 ± 0.8 (85.865)	1.2 ± 1.5 (113.136)	1 ± 1 (114.792)	5.977	2	.0504	na	na	na
	**PD**	**PSP‐P**	**PSP‐RS**	***χ*^2^**	***df***	***p***			
Prevalence of alexithymia status (%)	12.9%	54.5%	50%	21.027	2	<.0001*	na	na	na
Prevalence of anhedonia status (%)	75%	12.5%	12.5%	1.094	2	.5787	na	na	na

Note

Data represent mean ± *SD* (Mean Rank).

Abbreviations: TAS‐20, Toronto Alexithymia Scale‐20‐item; TAS‐20 F1, difficulty identifying feelings; TAS‐20 F2, difficulty describing feelings; TAS‐20 F3, externally oriented thinking; na, not applicable; PD, Parkinson's disease; PSP‐P, progressive supranuclear palsy with predominant parkinsonism; PSP‐RS, progressive supranuclear palsy–Richardson's syndrome.

* Significant after Bonferroni's correction for multiple comparisons (*n* = 4); *p* < .05/4 = *p* < .0125.

As to the ancillary analysis, we found differences in the frequency of depression diagnosis (minor depressive disorder and major depressive disorder) among the three groups (PD = 51; PSP‐P = 7, PSP‐RS = 8) (Tables 1 and 3). In particular, statistical significance was measured between PD and PSP‐P (*χ* ^2^ = 4.268; *df* = 1; *p* < .0388) and PD and PSP‐RS (*χ* ^2^ = 5.557; *df* = 1; *p* < .0184). Conversely, there was no difference between PSP‐P and PSP‐RS (*χ* ^2^ = 0.023; *df* = 1; *p* < .8789). Table 3 shows the alexithymic variables of the study population, split by the presence/absence of diagnosis of depression. Among patients with diagnosis of depression, statistically significant differences were found on TAS‐20 total and TAS‐20 F2 between PD and PSP‐RS, higher scores being measured in PSP‐RS group. Finally, there were no significant differences of TAS‐20 scores in nondepressed patients.

**Table 3:** Ancillary data on alexithymia in PD, PSP‐P, and PSP‐RS patients with and without depression diagnosis

Variables	PD (*n* = 51)	PSP‐P (*n* = 7)	PSP‐RS (*n* = 8)	*H*	*df*	*p* (Kruskal–Wallis)	Post hoc analysis (Mann–Whitney *U* test)		
							*p* PD vs. PSP‐P	*P* PD vs. PSP‐RS	*p* PSP‐P vs. PSP‐RS
Patients with diagnosis of depression									
TAS‐20 total	51.5 ± 12.6 (30.824)	54.8 ± 19.7 (36.000)	63.5 ± 8.4 (48.375)	5.914	2	.0520*	.5912	.0116	.5628
TAS‐20 F1	16.02 ± 6.7 (32.716)	17.1 ± 8.5 (35.357)	18.2 ± 8.7 (36.875)	0.398	2	.8196	na	na	na
TAS‐20 F2	15.1 ± 6.2 (31.059)	15.8 ± 7.7 (33.000)	21.1 ± 4.6 (49.500)	6.387	2	.0410*	.7837	.0124	.0826
TAS‐20 F3	20.4 ± 5.6 (31.059)	21.8 ± 11 (33.000)	24.1 ± 5.5 (44.938)	3.576	2	.1673	na	na	na

Variables	PD (*n* = 104)	PSP‐P (*n* = 4)	PSP‐RS (*n* = 4)	*H*	*df*	*p* (Kruskal–Wallis)	Post hoc analysis (Mann–Whitney *U* test)		
							*p* PD vs. PSP‐P	*p* PD vs. PSP‐RS	*p* PSP‐P vs. PSP‐RS
Patients without diagnosis of depression									
TAS‐20 total	43.5 ± 10.2 (54.635)	55.7 ± 17.9 (84.500)	50.7 ± 11.5 (77.000)	4.910	2	.0858	na	na	na
TAS‐20 F1 total	10.4 ± 4.2 (55.216)	15.2 ± 3.9 (91.500)	16.2 ± 16.6 (54.875)	4.819	2	.0899	na	na	na
TAS‐20 F2	12.5 ± 5.5 (55.529)	17.2 ± 8.5 (78.000)	14.0 ± 9.4 (60.250)	1.899	2	.3868	na	na	na
TAS‐20 F3	20.6 ± 5.6 (55.635)	23.2 ± 7.6 (70.500)	20.5 ± 10.7 (65.000)	1.091	2	.5795	na	na	na

Note

Data represent mean ± *SD* (Mean Rank).

Abbreviations: TAS‐20, Toronto Alexithymia Scale‐20‐item; TAS‐20 F1, difficulty identifying feelings; TAS‐20 F2, difficulty describing feelings; TAS‐20 F3, externally oriented thinking; na, not applicable; PD, Parkinson's disease; PSP‐P, progressive supranuclear palsy with predominant parkinsonism; PSP‐RS, progressive supranuclear palsy–Richardson's syndrome.

* Significant at *p* < .05.

## 4 DISCUSSION

To the best of our knowledge, reports on the prevalence and severity of alexithymia and anhedonia in subjects suffering from PSP variants are missing at present. Here, we provide evidence that alexithymia may burden PSP‐RS and PSP‐P since the early disease stage. The present findings may be relevant to the social, relational, and behavioral impairment experienced daily by PSP patients. Indeed, alexithymia impairs the ability to process and organize emotions and, therefore, may deconstruct the communicative and interactive relationships of patients. In particular, people with alexithymic status or symptomatology are unable to elaborate cognitively or conceptualize emotions by mental imaging or words.

Our results indicate that alexithymia distinguishes PSP from PD although it does not discern between the two PSP variants (PSP‐RS and PSP‐P; Table 2). This suggest that impairment in describing feeling (TAS‐20 F2) may be a distinct feature of PSP as compared to PD, possibly associated with the reduced ability of recognizing sad and happy facial expression by PSP as compared to PD patients (Pontieri et al., 2012). Indeed, patients with PSP‐RS show higher rate of alexithymic symptoms than those with PD. In particular, difficulty in describing feelings (TAS‐20 F2) may be a distinct feature of PSP‐RS in comparison to PD. The present finding, therefore, support the hypothesis that more severe alexithymic status and severity may represent a nonmotor signature of PSP, with potential relevance to precocious differential diagnosis.

Alexithymia in PSP‐RS is apparently linked to depression, as depressed PSP‐RS subjects showed a significantly higher score at TAS‐20 and TAS‐20 F2 than PD subjects (Table 3). Based on these findings, we speculate that the greater impairment of affective abilities in PSP as compared to PD patients may be related to the distinct pattern of frontal lobe and subcortical structures atrophy in the former (Brenneis et al., 2004; Piattella et al., 2015; Williams & Lees, 2009). In fact, prefrontal, limbic, and striatal abnormalities may contribute significantly to the derangement of effortful emotional regulatory processes and emotional processing (Assogna et al., 2011, 2016; Eack et al., 2016; Worker et al., 2014). Further, the difficulty in describing feeling (TAS‐F2) is a peculiar feature of PSP‐RS patients. This result is in line with the stronger verbal fluency impairment reported recently in PSP‐RS patients (Pellicano et al., 2017).

Interestingly, we could not find significant differences among the 3 groups as to hedonic state, as measured by the SHAPS score. This negative result may have been influenced by the dopaminergic therapy of our patients. Indeed, there is evidence for the specific involvement of ventral striatal dopaminergic projections in hedonic tone and for the inability of selective serotonin or noradrenaline reuptake inhibitors to reverse hedonic deficits (Carvalho et al., 2013). Alternatively, one should consider that the SHAPS is mostly focused on identifying consummatory and postconsummatory anhedonia rather than anticipatory anhedonia (Loas & Krystkowiak, 2010), the latter being preferentially affected in degenerative parkinsonian syndromes (Loas, Krystkowiak, & Godefroy, 2012).

Our study suffers from some limitations: our PSP patients were enrolled within Outpatient Services for Movement Disorders, suggesting a predominant motor phenotype of the disease; the results, therefore, may underestimate the prevalence and severity of affective impairment in early PSP cohorts. Moreover, we investigated only two PSP phenotypes, PSP‐RS as the classical presentation and PSP‐P being the variant more closely overlapping with idiopathic PD. Eventually, the small sample size of PSP‐RS and PSP‐P cohorts suggests confirmations of the present findings by further studies on larger series.

In conclusion, the results of this study show that PSP‐RS and PSP‐P patients exhibit stronger alexithymic features than PD since the early disease stage and that depression predicts alexithymia in PSP. These findings support the idea that impairment in processing and organizing emotional experiences mirrors a basic neurophysiological dysfunction occurring precociously in depressed PSP subjects. Therefore, the identification of alexithymia could orient the diagnosis toward PSP cases; particularly, difficulty in describing feelings could help to identify PSP‐RS patients. Our findings support the evidence that comprehensive psychiatric evaluations might be useful for diagnostic and therapeutic processes of PSP patients since the early stages of the disease.

## CONFLICT OF INTERESTS

The authors declare that there is no conflict of interests regarding the publication of this paper.

## AUTHOR CONTRIBUTIONS

Conception and design of the study: 1: Have made substantial contributions to conception and design, or acquisition of data, or analysis and interpretation of data; 2: Been involved in drafting the manuscript or revising it critically for important intellectual content; drafting the manuscript: 3: Given final approval of the version to be published. Each author should have participated sufficiently in the work to take public responsibility for appropriate portions of the content; 4: Agreed to be accountable for all aspects of the work in ensuring that questions related to the accuracy or integrity of any part of the work are appropriately investigated and resolved.

FA, CP, AS, BM, MP, FEP, and GS made substantial contributions to conception and design, acquisition of data, or analysis and interpretation of data; involved in drafting the manuscript or revising it critically for important intellectual content; gave final approval of the version to be published; and agree to be accountable for all aspects of the work in ensuring that questions related to the accuracy or integrity of any part of the work are appropriately investigated and resolved. LC, CS, LM, and CC involved in drafting the manuscript or revising it critically for important intellectual content; gave final approval of the version to be published; and agree to be accountable for all aspects of the work in ensuring that questions related to the accuracy or integrity of any part of the work are appropriately investigated and resolved. All authors approved the submitted version.

## Data Availability

The sociodemographic, clinical, neuropsychiatric, and cognitive data used to support the findings of this study are available from the corresponding author upon request.

## References

[brb31448-bib-0001] Assogna, F. , Cravello, L. , Caltagirone, C. , & Spalletta, G. (2011). Anhedonia in Parkinson's disease: A systematic review of the literature. Movement Disorders, 26(10), 1825–1834. 10.1002/mds.23815 21661052

[brb31448-bib-0002] Assogna, F. , Cravello, L. , Orfei, M. D. , Cellupica, N. , Caltagirone, C. , & Spalletta, G. (2016). Alexithymia in Parkinson's disease: A systematic review of the literature. Parkinsonism & Related Disorders, 28, 1–11. 10.1016/j.parkreldis.2016.03.021 27086264

[brb31448-bib-0003] Bagby, R. M. , Parker, J. D. , & Taylor, G. J. (1994). The twenty‐item Toronto Alexithymia Scale–I. Item selection and cross‐validation of the factor structure. Journal of Psychosomatic Research, 38(1), 23–32. 8126686 10.1016/0022-3999(94)90005-1

[brb31448-bib-0004] Bagby, R. M. , Taylor, G. J. , Parker, J. D. , & Loiselle, C. (1990). Cross‐validation of the factor structure of the Toronto Alexithymia Scale. Journal of Psychosomatic Research, 34(1), 47–51. 10.1016/0022-3999(90)90007-Q 2313613

[brb31448-bib-0005] Brenneis, C. , Seppi, K. , Schocke, M. , Benke, T. , Wenning, G. K. , & Poewe, W. (2004). Voxel based morphometry reveals a distinct pattern of frontal atrophy in progressive supranuclear palsy. Journal of Neurology, Neurosurgery and Psychiatry, 75(2), 246–249. PMC1738933 14742598

[brb31448-bib-0006] Carvalho, M. M. , Campos, F. L. , Coimbra, B. , Pego, J. M. , Rodrigues, C. , Lima, R. , … Salgado, A. J. (2013). Behavioral characterization of the 6‐hydroxydopamine model of Parkinson's disease and pharmacological rescuing of non‐motor deficits. Molecular Neurodegeneration, 8, 14 10.1186/1750-1326-8-14 23621954 PMC3653696

[brb31448-bib-0007] Colosimo, C. , Morgante, L. , Antonini, A. , Barone, P. , Avarello, T. P. , Bottacchi, E. , … Marconi, R. (2010). Non‐motor symptoms in atypical and secondary parkinsonism: The PRIAMO study. Journal of Neurology, 257(1), 5–14. 10.1007/s00415-009-5255-7 19669613

[brb31448-bib-0008] Eack, S. M. , Wojtalik, J. A. , Barb, S. M. , Newhill, C. E. , Keshavan, M. S. , & Phillips, M. L. (2016). Fronto‐limbic brain dysfunction during the regulation of emotions in schizophrenia. PLoS ONE, 11(3), e0149297. 26930284 10.1371/journal.pone.0149297 PMC4773075

[brb31448-bib-0009] Gelb, D. J. , Oliver, E. , & Gilman, S. (1999). Diagnostic criteria for Parkinson disease. Archives of Neurology, 56(1), 33–39. 10.1001/archneur.56.1.33 9923759

[brb31448-bib-0010] Hoglinger, G. U. , Respondek, G. , Stamelou, M. , Kurz, C. , Josephs, K. A. , Lang, A. E. , … Litvan, I. (2017). Clinical diagnosis of progressive supranuclear palsy: The movement disorder society criteria. Movement Disorders, 32(6), 853–864. 10.1002/mds.26987 28467028 PMC5516529

[brb31448-bib-0011] Litvan, I. , Agid, Y. , Calne, D. , Campbell, G. , Dubois, B. , Duvoisin, R. C. , … Zee, D. S. (1996). Clinical research criteria for the diagnosis of progressive supranuclear palsy (Steele‐Richardson‐Olszewski syndrome): Report of the NINDS‐SPSP international workshop. Neurology, 47(1), 1–9. 10.1212/wnl.47.1.1 8710059

[brb31448-bib-0012] Loas, G. , & Krystkowiak, P. (2010). The measurement of anhedonia in Parkinson's disease: Psychometric properties of the Snaith‐Hamilton Pleasure Scale (SHAPS) and the relevance to distinguish anticipatory and consummatory anhedonias. Movement Disorders, 25(4), 523–524. 10.1002/mds.22972 20108364

[brb31448-bib-0013] Loas, G. , Krystkowiak, P. , & Godefroy, O. (2012). Anhedonia in Parkinson's disease: An overview. The Journal of Neuropsychiatry and Clinical Neurosciences, 24, 444–451. 10.1176/appi.neuropsych.11110332 23224450

[brb31448-bib-0014] Parker, J. D. , Bagby, R. M. , & Taylor, G. J. (1991). Alexithymia and depression: Distinct and overlapping constructs? Comprehensive Psychiatry, 32(5), 387–394. 1743009 10.1016/0010-440x(91)90015-5

[brb31448-bib-0015] Pellicano, C. , Assogna, F. , Cellupica, N. , Piras, F. , Pierantozzi, M. , Stefani, A. , … Spalletta, G. (2017). Neuropsychiatric and cognitive profile of early Richardson's syndrome, Progressive Supranuclear Palsy‐parkinsonism and Parkinson's disease. Parkinsonism & Related Disorders, 45, 50–56. 10.1016/j.parkreldis.2017.10.002 29037499

[brb31448-bib-0016] Piattella, M. C. , Tona, F. , Bologna, M. , Sbardella, E. , Formica, A. , Petsas, N. , … Pantano, P. (2015). Disrupted resting‐state functional connectivity in progressive supranuclear palsy. American Journal of Neuroradiology, 36(5), 915–921. 10.3174/ajnr.A4229 25655870 PMC7990581

[brb31448-bib-0017] Pontieri, F. E. , Assogna, F. , Stefani, A. , Pierantozzi, M. , Meco, G. , Benincasa, D. , … Spalletta, G. (2012). Sad and happy facial emotion recognition impairment in progressive supranuclear palsy in comparison with Parkinson's disease. Parkinsonism & Related Disorders, 18(7), 871–875. 10.1016/jparkreldis.2012.04.023 22595619

[brb31448-bib-0018] Snaith, R. P. , Hamilton, M. , Morley, S. , Humayan, A. , Hargreaves, D. , & Trigwell, P. (1995). A scale for the assessment of hedonic tone the Snaith‐Hamilton Pleasure Scale. The British Journal of Psychiatry, 167(1), 99–103. 10.1192/bjp.167.1.99 7551619

[brb31448-bib-0019] Spalletta, G. , Fagioli, S. , Meco, G. , Pierantozzi, M. , Stefani, A. , Pisani, V. , … Assogna, F. (2013). Hedonic tone and its mood and cognitive correlates in Parkinson's disease. Depression and Anxiety, 30(1), 85–91. 10.1002/da.22036 23300113

[brb31448-bib-0020] Williams, D. R. , & Lees, A. J. (2009). Progressive supranuclear palsy: Clinicopathological concepts and diagnostic challenges. The Lancet Neurology, 8(3), 270–279. 10.1016/S1474-4422(09)70042-0 19233037

[brb31448-bib-0021] Worker, A. , Blain, C. , Jarosz, K. R. , Chauduri, K. R. , Barker, G. J. , Williams, S. C. , … Simmons, A. (2014). Cortical thickness, surface area and volume measures in Parkinson's disease, multiple system atrophy and progressive supranuclear palsy. PLoS ONE, 9(12), e114167 10.1371/journal.pone.0114167 25463618 PMC4252086

